# Predictive Modeling of Microbiome Data Using a Phylogeny-Regularized Generalized Linear Mixed Model

**DOI:** 10.3389/fmicb.2018.01391

**Published:** 2018-06-27

**Authors:** Jian Xiao, Li Chen, Stephen Johnson, Yue Yu, Xianyang Zhang, Jun Chen

**Affiliations:** ^1^Division of Biomedical Statistics and Informatics and Center for Individualized Medicine, Mayo Clinic, Rochester, MN, United States; ^2^School of Statistics and Mathematics, Zhongnan University of Economics and Law, Hubei, China; ^3^Department of Health Outcomes Research and Policy, Harrison School of Pharmacy, Auburn University, Auburn, AL, United States; ^4^Department of Statistics, Texas A&M University, College Station, TX, United States

**Keywords:** microbiome, phylogenetic tree, kernel method, generalized mixed model, predictive model

## Abstract

Recent human microbiome studies have revealed an essential role of the human microbiome in health and disease, opening up the possibility of building microbiome-based predictive models for individualized medicine. One unique characteristic of microbiome data is the existence of a phylogenetic tree that relates all the microbial species. It has frequently been observed that a cluster or clusters of bacteria at varying phylogenetic depths are associated with some clinical or biological outcome due to shared biological function (*clustered signal*). Moreover, in many cases, we observe a community-level change, where a large number of functionally interdependent species are associated with the outcome (*dense signal*). We thus develop “glmmTree,” a prediction method based on a generalized linear mixed model framework, for capturing clustered and dense microbiome signals. glmmTree uses the similarity between microbiomes, which is defined based on the microbiome composition and the phylogenetic tree, to predict the outcome. The effects of other predictive variables (e.g., age, sex) can be incorporated readily in the regression framework. Additional tuning parameters enable a data-adaptive approach to capture signals at different phylogenetic depth and abundance level. Simulation studies and real data applications demonstrated that “glmmTree” outperformed existing methods in the dense and clustered signal scenarios.

## 1. Introduction

The human microbiome, the collection of micro-organisms associated with the human body, has recently attracted substantial scientific interest due to its vital role in human health. For instance, the human gut microbiome contributes to nutrient metabolism, immune maturation and modulation, inflammatory cytokine production, and host gene regulation (Ahern et al., 2014; Schirmer et al., 2016; Pedersen et al., 2016; Fellows et al., 2018). Many diseases have been linked to dysbiosis of the microbiome ranging from metabolic disorders (e.g., obesity and type II diabetes) to autoimmune diseases (e.g., rheumatoid arthritis and multiple sclerosis) (Turnbaugh et al., 2009; Kinross et al., 2011; Cho and Blaser, 2012; Honda and Littman, 2012; Pflughoeft and Versalovic, 2012; Qin et al., 2012; Chen et al., 2016; Jangi et al., 2016). An abnormal microbiome has also been implicated in many cancer types such as colorectal, endometrial and esophageal cancers (Ahn et al., 2013; Bultman, 2014; Walther-Antonio et al., 2016; Peters et al., 2017), and a causal link has been emerging through deep mechanistic studies (Rubinstein et al., 2013; Bullman et al., 2017). In addition, the individual microbiomes may modulate drug pharmacokinetics and pharmacodynamics, contributing to drug response variations among individual patients (Haiser et al., 2014). Recently, the efficacy of cancer immune therapy has been shown to depend on the initial configuration of the gut microbiome (Gopalakrishnan et al., 2018; Matson et al., 2018; Routy et al., 2018). These findings open up the possibility of microbiome-based predictive medicine, where the microbiome data are used, potentially in conjunction with other clinic or omics data,to improve the prediction of relevant clinical outcomes.

A typical microbiome study involves collecting the microbiome samples, isolating all genomic DNA and sequencing the DNA using next-generation sequencing technologies. There are two main approaches to sequence the microbiome: gene-targeted sequencing and shotgun metagenomic sequencing (Kuczynski et al., 2011). In gene-targeted sequencing, a “fingerprint” gene that carries the taxonomic identity (e.g., 16S rRNA gene) is amplified and sequenced, while in shotgun metagenomic sequencing all genomic DNA is sequenced. Although shotgun metagenomics can profile both the taxonomic and functional content of the microbiome, the targeted approach has been more routinely employed to study the microbiome due to its lower cost and established bioinformatics pipelines. In the targeted approach, the sequencing reads are usually first clustered into operational taxonomic units (OTUs) based on the sequence similarity, via either *de novo* clustering or comparing to a reference database of OTUs (Edgar, 2013; Chen W. et al., 2013; Chen X. et al., 2018; Rideout et al., 2014). These OTUs are assumed to represent biological species at a 97% similarity level. Recently, the concept of “amplicon sequence variant” (ASV) has been proposed with the aim to cluster the sequence reads into a finer taxonomic resolution without the need for a particular similarity cutoff (e.g., 97%) (Callahan et al., 2016, 2017). After the clustering process, the sequencing reads from a targeted sequencing study are usually summarized as a count (abundance) table of the detected OTUs/ASVs. These OTUs/ASVs are all phylogenetically related, and a phylogenetic tree that reflects the evolutionary relationship can be built based on their sequence divergence (Price et al., 2010). Closely related species usually have similar biological functions, and they are likely to be associated with the outcome simultaneously, forming “clustered signals” (Martiny et al., 2015). These clustered signals can appear at a varying phylogenetic depth, resulting in clusters of different sizes (e.g., phyla and genera are at deep and shallow phylogenetic depths respectively) (Garcia et al., 2014). Thus, the phylogenetic tree provides important prior knowledge about how these species are related, which can be used to improve the efficiency of statistical analyses. Indeed, incorporation of the phylogenetic tree in the analysis has been instrumental in revealing overall community structure, identifying covariate-associated bacteria and improving the power of microbiome-wide testing (Purdom, 2011; Chen et al., 2012; Chen J. et al., 2013; Evans and Matsen, 2012; Xiao et al., 2017; Wang and Zhao, 2017).

To predict an outcome based on microbiome data, general-purpose machine learning methods, such as Random Forest and Support Vector Machine, as well as sparse regression models, such as Lasso (Tibshirani, 1996), MCP (Zhang, 1996), and Elastic Net (Zou and Trevor, 2005), have been applied (Knights et al., 2011; Statnikov et al., 2013; Pasolli et al., 2016). Although these methods are efficient in addressing the high dimensionality problem, they have a limited ability to exploit the phylogenetic structure of the microbiome data and hence may not be optimal if the signals are clustered. Many efforts have been attempted to incorporate the phylogenetic tree structure into prediction, mainly by imposing a novel phylogeny/tree-based smoothness penalty in penalized regression models. The phylogeny-based penalty encourages similar coefficients among species with respect to their phylogenetic relationship. For example, Tanaseichuk et al. (2014) used a tree-guided penalty to incorporate such structure into a penalized logistic regression framework. Chen et al. (2015) proposed a tree-based Laplacian penalty, in addition to a sparse penalty, for both classification and regression of microbiome data. These methods favor sparse and clustered signals due to their inherent sparsity assumption. However, a community-level change has frequently been observed in many physiological or pathophysiological states (Jernberg et al., 2010; Koenig et al., 2011; Milani et al., 2016), where a large number of functionally dependent species in the community are jointly associated with the outcome (“dense signal”). The “dense” signal is usually the consequence of the perturbation of the underlying microbial network, where species interact with each other to maintain a steady state (Faust and Raes, 2012). In such scenarios, although each species may have a weak effect on the outcome, the joint effects of all species may be strong. Thus, the sparsity assumption may not be desirable for “dense” microbiome signals.

In this work, we develop “glmmTree,” a predictive method based on a generalized mixed model framework, for capturing clustered and dense microbiome signals. To exploit the potential phylogenetic relatedness among species, the coefficients of the species are modeled as random with the correlation structure defined based on the phylogenetic tree. Other predictive variables (e.g., age, sex) are assumed to have fixed effects. One tuning parameter in the phylogeny-induced correlation structure allows detecting signals at various phylogenetic depths, and another tuning parameter facilitates differential weighting according to the species abundances as well as capturing certain non-linear relationships. Simulation studies and real data applications demonstrate that “glmmTree” outperforms existing methods in clustered and dense-signal scenarios.

## 2. Methods

### 2.1. A phylogeny-induced correlation structure among OTUs

Before we develop the predictive model for microbiome data, we first introduce a phylogeny-induced correlation structure among OTUs based on an evolutionary model. We use the term “OTU” throughout to represent a basic analysis unit. Assume that we have *p* OTUs on a phylogenetic tree and the patristic distance between OTU (i.e., the length of the shortest path linking OTU *i* and *j* on the tree) is denoted as *d*_*ij*_, the correlation of the traits between OTU *i* and *j* can be modeled using the following trait evolutionary model (Martins and Hansen, 1997).

(1) $$\begin{array}{r} C_{ij}\left(\rho \right)=e^{-2\rho d_{ij}},i,j=1,\ldots ,p. \end{array}$$

The parameter ρ ∈ (0, ∞) characterizes the evolutionary rate. If ρ = 0, then *C*_*ij*_ = 1, ∀*i, j*, indicating that all the traits are the same and there is no evolution at all. If ρ → ∞, then *C*_*ij*_ → 0, ∀*i, j*, indicating that the evolution is so fast that there is no correlation among the OTUs. In such case, the tree is not informative. Alternatively, ρ can be interpreted as a parameter that controls the phylogenetic depth at which the OTUs are grouped: larger ρ (smaller *C*_*ij*_) groups OTUs into clusters at a lower phylogenetic depth (a cluster is defined as a group of highly correlated OTUs). When ρ → ∞, there is no grouping of the OTUs. Conceptually, the phylogenetic grouping via ρ has a similar effect as taxonomic grouping, where OTUs at different taxonomic ranks (e.g., phylum, class, order, family, genus) are grouped according to their taxonomy. Compared to taxonomic grouping, the phylogenetic grouping circumvents the difficulty of the uncertainty in taxonomy assignments and achieves far more levels of granularities by adjusting ρ.

As the square root of the phylogenetic distance *d*_*ij*_ is of Euclidean nature (de Vienne et al., 2011), C(ρ) = (*C*_*ij*_(ρ))_*p*×*p*_ is positive definite by Bochner's theorem. In the proposed method, we recommend using $e^{-2\rho d_{ij}^{2}}$ to achieve an even better signal-grouping effect. Although the positive definiteness of C(ρ) is no longer theoretically guaranteed, it is positive definite or close to positive definite for most applications. In case of non-positive definiteness, we can perform positive definiteness correction (Higham, 2002).

### 2.2. glmmTree: a Generalized Linear Mixed Model based on a phylogenetic Tree

We assume that there are *n* samples with the abundances of *p* OTUs being profiled. For the *i*th sample, let *y*_*i*_ denote the outcome variable of interest, which can be binary or continuous (e.g., disease status, or body mass index), $z_{i}={\left(z_{i1},z_{i2},\ldots ,z_{ip}\right)}^{T}$ denote the normalized abundance vector of *p* OTUs (i.e., counts divided by the library size) for sample *i*, and $x_{i}={\left(x_{i1},x_{i2},\ldots ,x_{iq}\right)}^{T}$ be the *q* × 1 vector for covariates such as gender, age and other environmental or clinical variables that have predictive values. The goal is to predict *y*_*i*_ by ***z***_*i*_ and ***x***_*i*_.

For a continuous outcome variable, we use the linear mixed model (LMM) to build the prediction model

(2) $$\begin{array}{r} \begin{array}{c} y_{i}=\beta_{0}+x_{i}^{T}\beta_{1}+f{\left(z_{i};\gamma \right)}^{T}b+\epsilon_{i}\\ b\sim N\left(0,\sigma_{b}^{2}\text{C}\left(\rho \right)\right),\;\epsilon_{i}\sim N\left(0,\sigma_{\epsilon }^{2}\right), \end{array} \end{array}$$

and, for a binary outcome variable, we use the generalized linear mixed model (GLMM)

(3) $$\begin{array}{r} \begin{array}{c} \text{logit}\left(E\left(y_{i}\right)\right)=\beta_{0}+x_{i}^{T}\beta_{1}+f{\left(z_{i};\gamma \right)}^{T}b\\ b\sim N\left(0,\sigma_{b}^{2}\text{C}\left(\rho \right)\right), \end{array} \end{array}$$

where β_0_ is an intercept and $\beta_{1}={\left(\beta_{1},\beta_{2},\ldots ,\beta_{q}\right)}^{T}$ is a *q* × 1 vector of fixed effect regression coefficients for the *q* covariates, ϵ_*i*_ is the random error, $b={\left(b_{1},\ldots ,b_{p}\right)}^{T}$ is a *p* × 1 vector of random effect regression coefficients, C(ρ) = (*C*_*ij*_(ρ))_*p*×*p*_ is the phylogeny-induced correlation structure defined in the previous section, and $f\left(z_{i};\gamma \right)={\left(f\left(z_{i1};\gamma \right),\ldots ,f\left(z_{ip};\gamma \right)\right)}^{T}$ denotes some component-wise transformation of the abundance vector with the parameter γ allowing more modeling capability.

There are two advantages assuming the OTU effects ***b*** as random. Firstly, as the sample size is typically smaller than the number of OTUs (*p* > *n*), treating ***b*** as fixed effects will lead to overfitting on the training data and poor generalization on the test data. To improve the generalizability of the predictive model, the regression coefficients ***b*** need to be regularized. We thus put some distributional assumption on ***b*** and assume that ***b*** comes from a multivariate normal distribution with variance-covariance structure $\sigma_{b}^{2}\text{C}\left(\rho \right)$. The estimation procedure now switches from estimating *p* regression coefficients to estimating the variance component $\sigma_{b}^{2}$, which significantly reduces the number of parameters. Secondly, treating ***b*** as random effects provides the flexibility to incorporate prior structure information. For OTU data, the prior information is the phylogenetic relationship among OTUs, and closely related OTUs have a tendency to have similar effects. We incorporate such prior information using the phylogeny-induced correlation structure C(ρ). It should be noted that the ratio between $\sigma_{b}^{2}$ and $\sigma_{\epsilon }^{2}$ quantifies the joint (additive) OTU effects.

For the transformation function *f*(·), we propose using a power transformation, which is defined as

$$f(z_{ij},\gamma )=\{\begin{array}{l} z_{ij}^{\gamma }\;\;\;\;\;\;\;\;\;\;\;\;\;z_{ij}\neq 0\\ 0\;\;\;\;\;\;\;\;\;\;\;\;\;\text{otherwise} \end{array}$$

where γ is an unknown constant (γ ≥ 0). Similar to Box-Cox transformation (Sakia, 1992), it can potentially model a wide range of non-linear relationships between the OTU abundance and the outcome. This transformation takes into account the skewed OTU abundance distribution and allows differential weighting according to the abundance level. Smaller values of γ (e.g., 0.1) up-weight less abundant OTUs so that their effects will not be masked by those dominant OTUs when the signals are primarily in the less abundant OTU clusters. When γ approaches 0, the OTU abundance data become almost binary. In this case, only presence/absence of the OTU matters and these dominant OTUs contribute little to the outcome since they are present in most samples.

In the model, the regression coefficients β_0_ and **β**_1_, and the variance components $\sigma_{b}^{2},\sigma_{\epsilon }^{2}$ need to be estimated from the data. In principle, the parameters ρ and γ can also be estimated. However, in our application, we treat them as tuning parameters, and their optimal values are selected using cross-validation. We account for potential non-informativeness of the phylogenetic tree (i.e., signals are not clustered with respect to the tree) by including a very large value on the search grid of ρ.

Our phylogeny-based LMM or GLMM can be written in another form,

(4) $$\begin{array}{r} \begin{array}{c} g\left(E\left(y_{i}\right)\right)=\beta_{0}+x_{i}^{T}\beta_{1}+h_{i}\\ h={\left(h_{1},h_{2},\ldots ,h_{n}\right)}^{T}\sim MVN\left(0,\sigma_{b}^{2}\text{K}\left(\gamma ,\rho \right)\right) \end{array} \end{array}$$

where *g*(.) is the link function, ***h*** are the aggregated OTU effect (overall microbiome effect) and K(γ, ρ) is a phylogeny-based kernel matrix by evaluating the kernel function

$$K(z_{i},z_{j};\gamma ,\rho )=f{\left(z_{i};\gamma \right)}^{T}\text{C}(\rho )f(z_{j};\gamma )$$

at all pairs of observations. The phylogeny-based kernel function *K*(·, ·;γ, ρ) quantifies the similarity between observations in terms of OTU abundance profile (“microbiome similarity”) while taking into account the phylogenetic tree structure. Similar ideas have been used to define ecological distances between microbiome samples such as the popular UniFrac distance (Lozupone and Knight, 2005). From (4), we can see that our model aims to predict the outcome based on the microbiome similarities while the tuning parameters γ, ρ are used to tailor the microbiome similarity measure to maximally reflect the outcome similarity. Since the microbiome similarity is calculated based on all OTUs, the model is expected to perform best when the signals are relatively dense, i.e., there are many outcome-associated OTUs.

Our model is closely related to the kernel machine-based semi-parametric regression model (KMR) (Liu et al., 2007, 2008)

(5) $$g(E(y_{i}))=\beta_{0}+x_{i}^{T}\beta_{1}+h_{K}(z_{i}),$$

where the covariate effect is modeled parametrically, and the overall OTU effect is modeled non-parametrically through an unknown function *h*_*K*_(·) that belongs to a Reproducing Kernel Hilbert Space (RKHS) $H_{K}$ generated by the kernel function *K*(·, ·). It turns out that the penalized likelihood estimation for KMR is equivalent to the maximum likelihood estimation in GLMM.

### 2.3. Model estimation

The parameter ρ, controlling the evolutionary rate, and the parameter γ, controlling the non-linear effect, are treated as known in model estimation. For a continuous outcome, the LMM is fitted using the restricted maximum likelihood estimation method (RMLE) as described in Kang et al. (2008). Newton-Raphson algorithm can be used to find the optimal solution. For a binary outcome, the GLMM is fitted by the penalized quasi-likelihood (PQL) method proposed by (Breslow and Clayton, 1993). PQL approximates the high-dimensional integration over ***b*** using the Laplace approximation, and the approximated likelihood function has that of a Gaussian distribution. Therefore, the PQL estimate can be obtained by fitting a series of LMMs. Details of the algorithms can be found in the Supplementary Note.

### 2.4. Prediction of new observations

Once the model is fitted based on the training dataset, prediction can be made on the new observations. In this section, we describe in detail how to predict the outcome of new observations to provide more insights into our predictive model. Suppose we have *n*_*tr*_, *n*_*te*_ observations in the training and test dataset respectively. Let ***y***_*tr*_, ***y***_*te*_ be the outcome vectors of the training and test dataset respectively, X_*tr*_, X_*te*_ be the design matrices for fixed effects including the intercepts and Z_*tr*_, Z_*te*_ be the OTU abundance matrices. We further denote $K_{tr}=f\left(Z_{tr};\gamma \right)C\left(\rho \right)f{\left(Z_{tr};\gamma \right)}^{T},K_{te}=f\left(Z_{te};\gamma \right)C\left(\rho \right)f{\left(Z_{te};\gamma \right)}^{T}$ and $K_{tr,te}=f\left(Z_{tr};\gamma \right)C\left(\rho \right)f{\left(Z_{te};\gamma \right)}^{T}$, which are the kernel matrices describing the microbiome similarities. We focus on the prediction of a continuous outcome and the prediction of a binary outcome can similarly be made based on the working LMM model at the convergence of the PQL algorithm.

Based on (4), the joint distribution of ***y***_*tr*_ and ***y***_*te*_ can be written as

(6) $$\left(\begin{array}{c} y^{tr}\\ y^{te} \end{array}\right)\sim \text{MVN}\left\{\left(\begin{array}{c} {\text{X}}_{tr}\beta \\ {\text{X}}_{te}\beta \end{array}\right),\;\left(\begin{array}{cc} \Sigma_{tr} & \Sigma_{tr,te}\\ \Sigma_{te,tr} & \Sigma_{te} \end{array}\right)\right\},$$

where $\beta ={\left(\beta_{0},\beta_{1}^{T}\right)}^{T}$, $\Sigma_{tr}=\sigma_{b}^{2}{\text{K}}_{tr}+\sigma_{\epsilon }^{2}\text{I}$ and $\Sigma_{te}=\sigma_{b}^{2}{\text{K}}_{te}+\sigma_{\epsilon }^{2}\text{I}$ are variance-covariance matrices for training and test dataset respectively, and $\Sigma_{te,tr}=\Sigma_{tr,te}^{T}=\sigma_{b}^{2}{\text{K}}_{tr,te}$ is the covariance matrix between training and test dataset. From the linear model theory, the conditional distribution of ***y***_*te*_ on ***y***_*tr*_ is given by

(7) $$\begin{array}{c} (y_{te}|y_{tr})\sim \text{MVN}({\text{X}}_{te}\beta +\Sigma_{te,tr}\Sigma_{tr}^{-1}(y_{tr}-{\text{X}}_{tr}\beta ),\;\Sigma_{te}\\ -\Sigma_{te,tr}\Sigma_{tr}^{-1}\Sigma_{tr,te}). \end{array}$$

Thus, the prediction of ***y***_*te*_ can be obtained based on

$$\begin{array}{lll} {\tilde{y}}_{te} & = & E\left[y_{te}|y_{tr}\right]\\ = & {\text{X}}_{te}\beta +\Sigma_{te,tr}\Sigma_{tr}^{-1}\left(y_{tr}-{\text{X}}_{tr}\beta \right). \end{array}$$

Plugging in the estimates of **β**, $\sigma_{b}^{2}$ and $\sigma_{\epsilon }^{2}$ based on the training dataset, we obtain the final prediction as

$$\begin{array}{l} {\hat{y}}_{te}={\text{X}}_{te}\hat{\beta }+{\hat{\Sigma }}_{te,tr}{\hat{\Sigma }}_{tr}^{-1}\left(y_{tr}-{\text{X}}_{tr}\hat{\beta }\right). \end{array}$$

Note that the prediction formula can also be written in terms of the random effects ***b***:

$$\begin{array}{l} {\hat{y}}_{te}={\text{X}}_{te}\hat{\beta }+f\left(Z_{te};\gamma \right)\hat{b}, \end{array}$$

where $\hat{b}$ is the best linear unbiased predictor (BLUP), which is a smoothed estimate with respect to the phylogenetic tree (Supplementary Note).

The “glmmTree” software is available at “https://github.com/lichen-lab/glmmTree.”

## 3. Simulation studies

### 3.1. Simulation strategy

We carried out extensive simulations to evaluate the performance of glmmTree for both continuous and binary outcomes. For the continuous outcome, we simulated 100 independent samples in the training set and 200 independent samples in the test set. For the binary outcome, we simulated 50 cases and 50 controls in the training set, and 100 cases and 100 controls in the test set. We used a Dirichlet-multinomial distribution to simulate OTU counts and generated the outcome based on the abundances of several selected OTU clusters. To objectively evaluate our predictive model, we performed a parameter sweep and investigated the effect of the cluster size (phylogenetic depth), the number of clusters (signal density) and the abundance level of the clusters on the prediction performance. The simulation studies were aimed to reveal the scenarios under which our model performed favorably and also identify potential “blind spots” of our model.

#### 3.1.1. Simulating OTU abundance data

We generated the OTU counts using a Dirichlet-multinomial distribution with the parameters (the mean proportion vector and the dispersion parameter ϕ) estimated based on a real OTU dataset from a study of the microbiome of the human upper respiratory tract (Charlson et al., 2010; Chen and Li, 2013), which contains the counts of 778 OTUs from 60 samples, together with a phylogenetic tree describing the evolutionary relationship among the 778 OTUs. For each sample, the total read count was drawn from a negative binomial distribution with mean 5000 and dispersion 25. The OTU counts were normalized into OTU proportions (z) by dividing the total read counts.

#### 3.1.2. Constructing outcome-associated OTU clusters

The underlying relationship between the outcome and the microbiome is complex. The outcome-associated OTUs (“aOTUs”) can be clustered at different phylogenetic depths (deep or shallow), creating OTU clusters (“aClusters”) of different sizes. It is also possible that the aOTUs are simply not phylogenetically related. In such case, each aOTU constitutes an aCluster of size 1. The signal density (number of aClusters) can also vary depending on the outcome. Finally, aClusters can be abundant or rare since both rare and abundant taxa have been observed to associate with the outcome. We thus studied the effects of all these parameters in the simulation.

To construct aClusters with a different level of cluster size, signal density and abundance, 778 OTUs were first grouped into *m* clusters based on their patristic distances on the phylogenetic tree.

**Figure 1:**
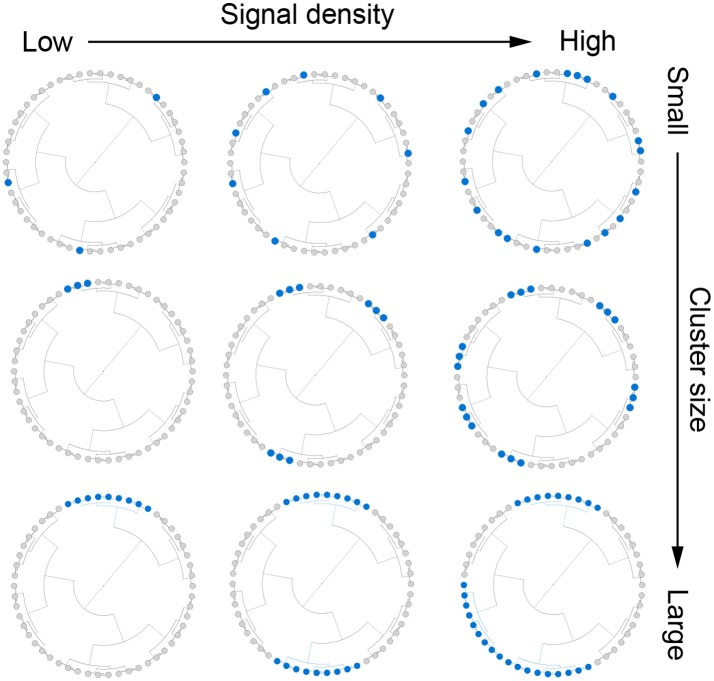
Illustration for the simulation strategy. We simulate outcome-associated OTU clusters (aClusters) of different cluster size (**top** to **bottom**) and signal density (**left** to **right**). We also vary the abundance level of the aClusters (not shown).

We assumed that there were *m*_*c*_ (*m* × *s%*) aClusters and *s%* represents the signal density. For given *m* and *m*_*c*_, we chose aClusters of different abundance level (*a*). The simulation strategy is illustrated in Figure 1 and the detailed settings for cluster size, signal density and abundance are presented below:

- **Cluster size** (**m**):
  The 778 OTUs were partitioned into *m* clusters using the partitioning-around-medoids (PAM) algorithm based on the patristic distances among OTUs (Chen et al., 2012). We considered *m*∈(10, 100, 778), representing large, medium and small OTU clusters, and aClusters were selected from these OTU clusters. Note that when *m* = 778, the aOTUs are not phylogenetically related and the phylogenetic tree is not informative for prediction.
- **Signal density** (**s**%): We selected *s%*∈(10%, 20%, 40%) for *m* = 10, *s%*∈(1%, 5%, 25%) for *m* = 100 and *s%* ∈ (1%, 5%, 30%) for *m* = 778 to represent low, medium and high signal density respectively. The number of aClusters *m*_*c*_ was taken to be the integer part of *m* × *s%*.
- **Abundance** (***a***): Given *m* and *m*_*c*_, we had (mmc) choices of aClusters. To obtain low, medium and high abundance level, we randomly picked *m*_*c*_ clusters from *m* clusters 1000 times and recorded their cumulative abundances *a*_*t*_ (*t* = 1, …, 1000). We chose *m*_*c*_ aClusters of high, medium and low abundance with abundance max(*a*_*t*_), median(*a*_*t*_), min(*a*_*t*_), *t* = 1, …, 1000, respectively.

#### 3.1.3. Generating the outcome based on the abundance of AClusters

Denote *C*_*l*_ as the set containing the indices of the *l*th aCluster, *l* ∈ {1, …, *m*_*c*_}, and η_*i*_ be the expected outcome value for sample *i*. We first generated η_*i*_ based on the following linear relationship

(8) $$\begin{array}{r} \begin{array}{c} \eta_{i}=\beta_{0}+\overset{m_{c}}{\underset{l\;=\;1}{\sum }}\left(\underset{k\;\in C_{l}}{\sum }z_{ik}\right)b_{l}\\ b_{l}\sim N\left(0,\sigma_{b}^{2}\right) \end{array} \end{array}$$

For a continuous outcome,

(9) $$\begin{array}{r} y_{i}=\eta_{i}+\epsilon_{i},\;\epsilon_{i}\sim N\left(0,\sigma_{\epsilon }^{2}\right) \end{array}$$

For a binary outcome,

(10) $$\begin{array}{r} \begin{array}{c} \pi_{i}=\frac{e^{\eta_{i}}}{1+e^{\eta_{i}}}\\ y_{i}\sim Bernoulli\left(\pi_{i}\right) \end{array} \end{array}$$

Note that we assigned the same coefficient for OTUs within the same cluster to create clustered signals. The variance $\sigma_{b}^{2}$ can be adjusted to control the signal-to-noise ratio. Without loss of generality, $\sigma_{b}^{2}$ was set to be 2 for the continuous outcome and 4 for the binary outcome. The error variance $\sigma_{\epsilon }^{2}$ for the continuous outcome was chosen to be $\frac{1}{4}\text{var}\left(Zb\right)$ so that the OTUs jointly explain 80% of the outcome variability.

To study the prediction performance under potential non-linearity, we also simulated non-linear relationships, where we use *f*(*z*_*ik*_) instead of *z*_*ik*_ to generate the outcome. We specifically investigated when $f\left(z_{ik}\right)=z_{ik}^{0.5}$, which attenuates the effect of highly abundant OTUs, and *f*(*z*_*ik*_) = 1(if *z*_*ik*_ ≠ 0), which represents the scenario where only the presence/absence of the OTU affects the outcome.

### 3.2. Competing methods, model selection and evaluation

#### 3.2.1. Competing methods

We compared glmmTree to Lasso, MCP and Elastic Net (Enet), three sparse regression models with no consideration of the phylogenetic structure. Particularly, Elastic Net encourages the data-driven smoothing via *L*_2_ penalty. We also compared glmmTree to a phylogeny-constrained sparse regression model (Chen et al., 2015) as a representative of tree-structure penalized regression models. The method uses the same phylogeny-induced correlation structure as in glmmTree but encourages the phylogeny-driven smoothing based on the inverse correlation matrix instead of the usual Laplacian matrix. We thus termed it Sparse Inverse Correlation Shrinkage method (SICS). Besides those sparse regression models, we also compared glmmTree to Random Forest (RF), which has been demonstrated a superior prediction performance in various microbiome datasets. Finally, we compared to a regular kernel-based GLMM (glmmTree.Reg) to evaluate the benefit of exploiting the phylogenetic tree in prediction.

#### 3.2.2. Model selection and evaluation

For glmmTree, the tuning parameters (γ, ρ) are used to control the phylogenetic depth and non-linear effect and need to be tuned. We searched ρ on the grid $\underset{11}{\underset{︸}{\left\{0,2^{-5},2^{-4},2^{-3},\cdots \;,2^{4},2^{5}\right\}}}$ while γ was tuned on the grid $\underset{12}{\underset{︸}{\left\{0,0.01,0.1,0.3,0.5,0.7,\ldots ,1.9\right\}}}$. glmmTree.Reg was achieved by fixing ρ at a very large value (10^4^). The details of specific software packages used and their parameter settings for competing methods are shown in Box 1.

Box 1 **Tuning parameter settings in different methods.**

- Lasso: *glmnet* R package, all parameters were set as the default.
- Elastic Net: *glmnet* R package, all parameters were set as the default.
- MCP: *ncvreg* R package, all parameters were set as the default
- SICS: *glmgraph* R package, the search grid for ρ was the same as glmmTree, the tuning parameter for the smoothness penalty was selected from $\underset{11}{\underset{︸}{\left\{0,2^{-5},2^{-4},2^{-3},\cdots \;,2^{4},2^{5}\right\}}}$, other parameters were set as default.
- Random Forest: *randomForest* R package, parameters were set as default.

Tuning parameter selection was based on five-fold cross-validation (CV), where the training samples were randomly divided into five folds with four folds used for model fitting and the remaining one for calculating some CV criterion. We used PMSE (Predicted Mean Square Error) as the CV criterion for a continuous outcome and AUC (Area Under the Curve) for a binary outcome. Once the optimal values of the tuning parameters were selected, we fit the model using all training sample (*n* = 100) and then evaluated the prediction performance on the test dataset (*n* = 200). Although we used PMSE and AUC for tuning parameter selection, we focused on *R*^2^, which quantifies the correlation between the predicted outcome and the observed outcome and ranges from 0 (no correlation) to 1 (perfect correlation), to evaluate the prediction performance. Specifically, for a continuous outcome, *R*^2^ is defined as

$${\text{R}}^{2}=\frac{{\left\{\sum_{i\;=\;1}^{n_{te}}\left({ŷ}_{te,i}-{\bar{ŷ}}_{te}\right)\left(y_{te,i}-{\bar{y}}_{te}\right)\right\}}^{2}}{\sum_{i\;=\;1}^{n_{te}}{\left({ŷ}_{te,i}-{\bar{ŷ}}_{te,}\right)}^{2}\sum_{i\;=\;1}^{n_{te}}{\left(y_{te,i}-{\bar{y}}_{te}\right)}^{2}},$$

where $\bar{ŷ},ȳ$ are the sample means. For the binary-version *R*^2^, we substitute ŷ_*te,i*_ with the predicted probability ${\hat{P}}_{te,i}$. Each simulation was repeated 50 times and means and standard errors were reported.

### 3.3. Simulation results

#### 3.3.1. Results for the continuous outcome.

**Figure 2:**
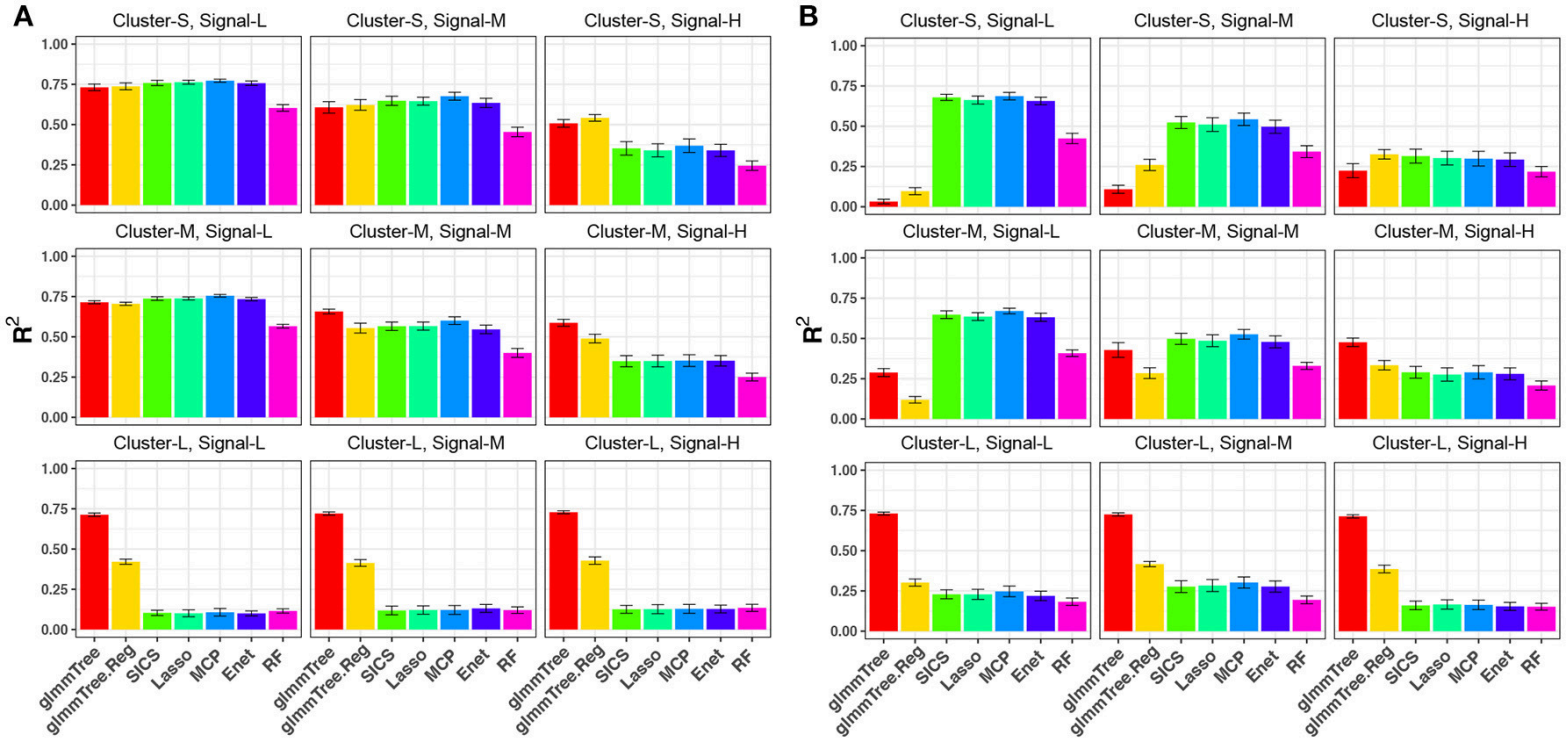
*R*^2^ for continuous-outcome simulations across different levels of cluster size and signal density. The abundance of associated OTU clusters is chosen to be high **(A)** and medium **(B)**. Cluster-S, -M, and -L represent small, medium and large clusters, and Signal-L, -M, and -H represent low, medium and high signal density, respectively.

We first evaluated the performance of different methods across different cluster sizes and signal densities when the abundance of the aClusters was high (Figure 2). We observed a general decrease in performance for all methods when the signal density increased. This trend is explained by a result of decreasing individual effects as we increased the number of aOTUs since we fixed the percentage of variability explained by OTUs (80%) across parameter settings. The reduction in individual effects was unfavorable for all methods. When the aCluster was large, i.e., the signals were highly clustered, glmmTree outperformed other methods substantially. Particularly, glmmTree had a clear advantage over glmmTree.Reg, which did not account for the phylogenetic structure, indicating the benefit of using phylogenetic information to improve prediction. It was also significantly better than the sparse regression methods and RF across different levels of signal density. The unfavorable performance of these sparse regression methods was due to the weak individual effects of these aOTUs in the large cluster. In such “many OTUs, weak effects” scenario, sparse regression methods tended to have a low sensitivity and specificity to identify these aOTUs, which led to poor prediction performance. As the cluster size decreased, the phylogenetic signal became weaker, and the difference of performance between glmmTree and other methods diminished accordingly. However, glmmTree still performed better than sparse regression methods when the signal was dense. This was due to the fact that glmmTree did not assume sparsity in the model, and when the signal became dense, the irrelevant OTUs did not seriously corrupt the overall microbiome similarity, upon which the glmmTree was based. It should be noted that glmmTree and glmmTree.Reg had performance similar to those sparse regression methods in their most unfavorable setting, where a small number of phylogenetically non-related OTUs were associated with the outcome (Figure 2A, upper left). The comparable performance is explained by the high abundance of the aOTUs, which dominated those rare and less abundant OTUs in determining the microbiome similarity.

As we decreased the abundance of the aClusters to be “medium” (Figure 2B), glmmTree still excelled in highly clustered signals across different signal densities, but its prediction performance deteriorated significantly as the signal density became lower and the size of aCluster became smaller. When the signals were not phylogenetically related (Figure 2B, top row), sparse regression models and RF performed better than glmmTree. As these phylogenetically non-related signals grew more sparse, glmmTree had very low predictive power. A similar trend was observed when the abundance of aClusters was “low” (Figure S1). In this scenario, the phylogeny-regularized sparse regression method (SICS) outperformed the other sparse regression methods. In summary, no methods dominates in all settings and glmmTree has a performance edge over other competing methods when the signal is *dense, clustered* and/or *abundant*.

**Figure 3:**
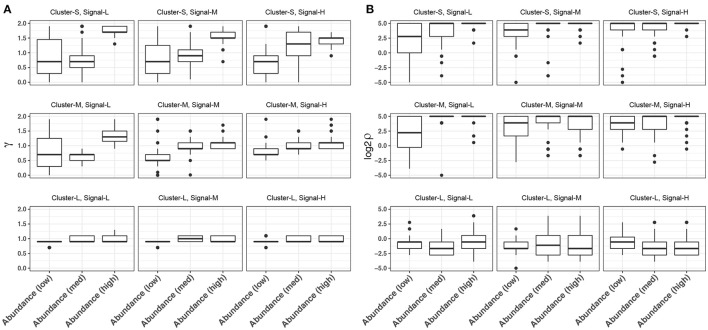
Distribution of the selected tuning parameter γ **(A)** and ρ **(B)** across different levels of cluster size, signal density and abundance for continuous-outcome simulations. Cluster-S, -M, and -L represent small, medium and large clusters, and Signal-L, -M, and -H represent low, medium and high signal density, respectively.

In glmmTree, we included two tuning parameters γ, which up-weights or down-weights the effect of abundant OTUs, and ρ, which controls the phylogenetic depth of the signal. These two tuning parameters are used to exploit various signal structures for microbiome data. It is interesting to observe the patterns of the selected values across simulation settings. We plotted the distribution of selected γ and ρ values over the fifty simulation runs across different levels of cluster size, signal density and abundance for the continuous outcome (Figure 3). As expected, smaller values of γ tended to be selected for “low-abundance” scenarios, where the outcome was associated with less abundant aClusters. Smaller γ values up-weighted the effects of less abundant OTUs and hence amplified their weak signals (Figure 3A). γ had the stronger impact when the phylogenetic signal was weak (i.e., the OTUs were less phylogenetically related). On the other hand, smaller ρ values were selected for larger clusters, where the signals were at a deeper phylogenetic depth (Figure 3B). Therefore, the inclusion of these two tuning parameters improved the model flexibility.

To study the robustness of glmmTree to tree mis-specification, we generated “noisy” trees by randomly permuting different percentages of the rows/columns of the tree-induced distance matrices. As we increased the percentage from 25 to 75%, the performance of glmmTree decreased accordingly, but it was still more powerful than glmmTree.Reg, which did not use tree information (Figure S2). As the tuning parameter ρ approaches infinity, glmmTree is reduced to glmmTree.Reg. Therefore, the performance of glmmTree is expected to be close to glmmTree.Reg when the tree is severely mis-specified. We next studied the performance of glmmTree under much lower percentages of variability explained by OTUs (50% and 33%). As we lowered the signal-noise-ratio (SNR), the performance of all methods deteriorate but the same trend has been observed as in the high SNR scenario (Figure S3).

#### 3.3.2. Results for the binary outcome.

**Figure 4:**
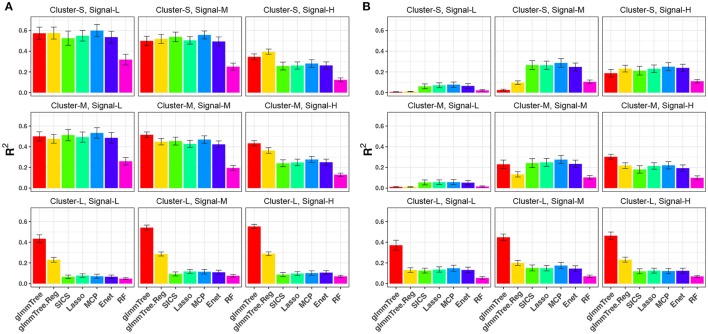
*R*^2^ for binary-outcome simulations across different levels of cluster size and signal density. The abundance of associated OTU clusters is chosen to be high **(A)** and medium **(B)**. Cluster-S, -M, and -L represent small, medium and large clusters, and Signal-L, -M, and -H represent low, medium and high signal density, respectively.

We repeated the same simulations for the binary outcome and present the results in Figure 4 and Figure S4. Compared to the continuous outcome-based simulations, the performance for all methods deteriorated faster when the aClusters became less abundant and more sparse. Nevertheless, a similar trend persisted: glmmTree had the best performance under clustered and dense signals, and abundant aClusters further improved its performance.

#### 3.3.3. Accommodation for non-linear signals

**Figure 5:**
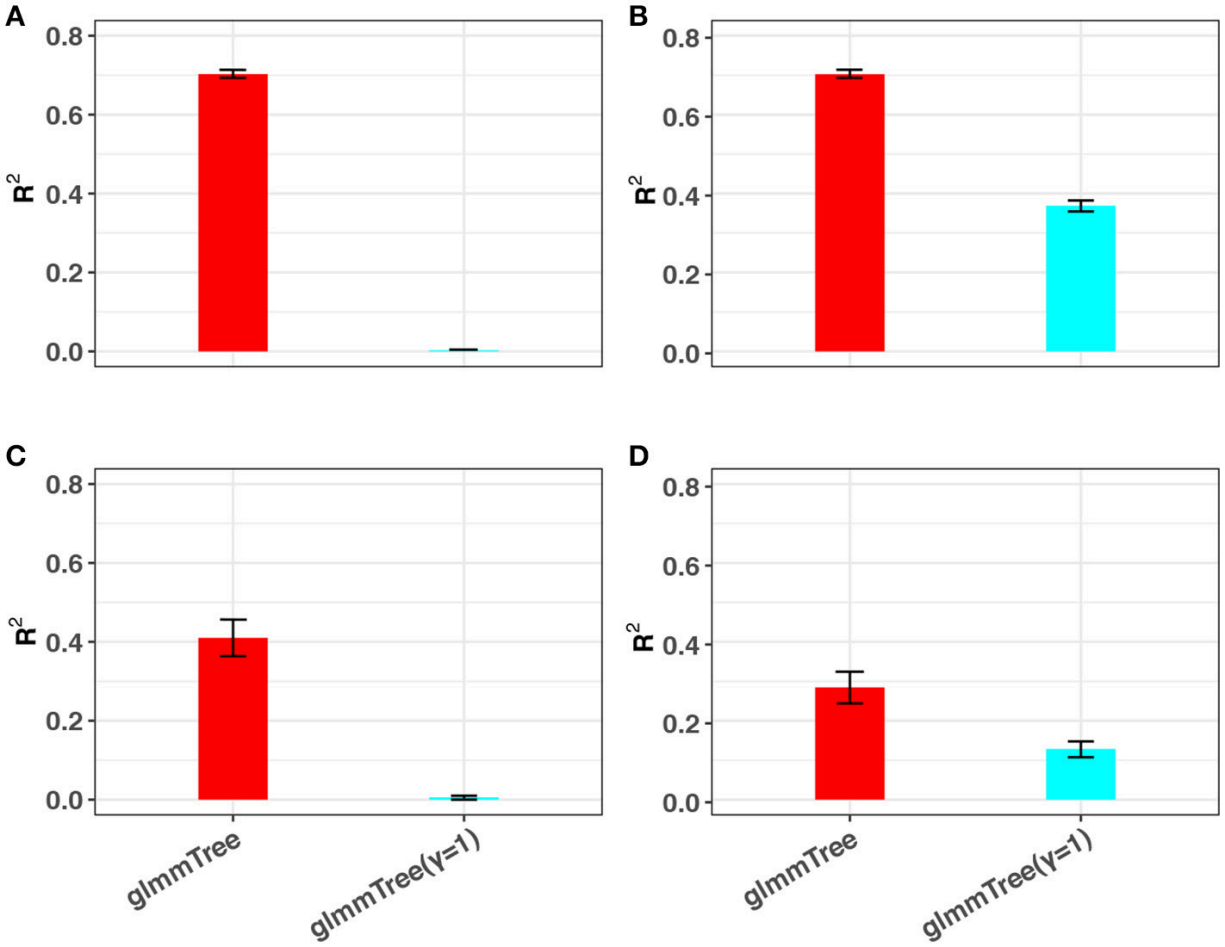
The ability of glmmTree to capture non-linear effects through the tuning parameter γ. glmmTree with tunable γ (red) is compared to glmmTree with fixed γ = 1 (blue). *R*^2^ is used to evaluate the performance for continuous **(A,B)** and binary **(C,D)** outcomes when the outcome is generated based on OTU presence/absence **(A,C)** and square-root transformed OTU abundances **(B,D)**.

The conclusions in the previous simulations were based on linear signals. Since the relationship between the microbiome and the outcome is very complex, traditional linear models may fail to capture non-linear microbiome effects. Besides the differential weighting function, the tuning parameter γ can also accommodate a wide range of non-linear effects. To illustrate this point, we performed additional simulations based on non-linear signals and compared the prediction performance to glmmTree with a fixed gamma value (γ = 1). Specifically, we investigated two types of non-linear relationships, in which the outcome was generated based on (1) the OTU presence/absence and (2) square-root transformed OTU abundances, respectively. Without loss of generality, we set the scenario to be high abundance, large cluster and low signal density. The simulation results are presented in Figure 5. Clearly, glmmTree achieved a significantly higher *R*^2^ than glmmTree without γ tuning in both non-linear scenarios for both continuous and binary outcomes. When the outcome depended on the OTU presence/absence, glmmTree without γ tuning was powerless: the *R*^2^ was close to 0. In contrast, glmmTree with γ tuning performed substantially better since γ was usually tuned to be close to 0 to accommodate such non-linearity. When the outcome depended on the square-root transformed OTU abundances, glmmTree without γ tuning achieved some predictive power, but was still much less powerful than glmmTree with γ tuning. Therefore, glmmTree can also capture non-linear signals with the imbedded power transformation.

## 4. Application of glmmTree to predicting chronological age based on the human gut microbiome

We applied glmmTree to a study investigating how the gut microbiome differs across age and geography (Yatsunenko et al., 2012). The study consisted of 531 individuals, among which 115 individuals were from Malawi, 100 individuals were from Venezuela, and 316 individuals were from the USA. The gut microbiota of these individuals was profiled using 16S rRNA gene targeted sequencing. The dataset was available for download from Qiita (https://qiita.ucsd.edu/) with study ID 850, where the sequence data was processed by the QIIME pipeline (reference-based approach). A total of 14,170 OTUs were produced for this dataset. To demonstrate the performance of glmmTree, we used the 316 individuals from the USA for age prediction.

The complexity of the real data required us to properly normalize, transform and filter the data before applying various predictive tools. Let (*c*_*ij*_)_*p*×*n*_ be the observed count matrix. We carried out a series of pre-processing steps before applying various prediction methods:

1. Sample filtering to remove outlier samples. We calculated the Bray-Curtis distance between samples. Denote *d*_*jk*_ the distance between sample *j* and *k*. For each sample *j*, we calculated the median distance from sample *j* to other samples, denoted as *m*_*j*_ = *Median*_*k*≠*j*_(*d*_*jk*_). An outlier index *o*_*j*_ for sample *j* was defined as *o*_*j*_ = *m*_*j*_/*Median*_*k*_(*m*_*k*_). We removed samples with *o*_*j*_ > 2 (8 samples removed).
2. OTU filtering to remove less informative and noisy OTUs and reduce dimensionality. We imposed two filters: (1) OTU prevalence < 10%, and (2) Median non-zero counts < 10.
3. Normalization to address variable library sizes. We used GMPR normalization, which is developed specifically for zero-inflated count data (Chen L. et al., 2018). For each sample, we calculated a GMPR size factor *s*_*j*_ and the normalized counts were then divided by *s*_*j*_. The normalized counts are denoted as ${\left({\tilde{c}}_{ij}\right)}_{p\times n}$.
4. Winsorization to replace outlier counts. For each taxon *i*, we calculated the 97% quantile $q_{i}^{0.97}$ based on ${\tilde{c}}_{ij}\left(j=1\cdots n\right)$, and replaced ${\tilde{c}}_{ij}>q_{i}^{0.97}$ with $q_{i}^{0.97}$. This procedure has shown to be effective in reducing false positives in the context of differential abundance analysis (Chen J. et al., 2018).
5. Transformation to reduce the influence of highly abundant taxa counts. We used the commonly used square-root transformation.
6. We further used square-root transformation on the continuous age variable to better capture the underlying relationship.

These proprocessing steps were used to make the microbiome data more amenable to predictive modeling, and could improve the performance of sparse regression methods such as Lasso (Figure S5). After the processing steps, we were left with 308 individuals and 1087 OTUs. We first evaluated the prediction performance by treating age as a continuous outcome. To demonstrate the performance with binary outcomes, we classified the individuals into three age groups: baby (age ≤ 3 years, *n* = 54), child (3 < age < 18 years, *n* = 125) and adult (age ≥ 18 years, *n* = 129), and evaluated the prediction performance based on the baby and child age group. The guidance of the group division and choice was based on the observation that the microbiome change begins to slow down after three years old, and the child microbiome is more similar to the adult microbiome (Yatsunenko et al., 2012). We included the prediction of baby vs. child in the main text and the prediction of child vs. adult in the Supplementary File.

We compared glmmTree to SICS, Lasso, MCP, Elastic Net and Random Forest. Tuning parameter selection was based on cross-validation (CV) as in the simulation.

**Figure 6:**
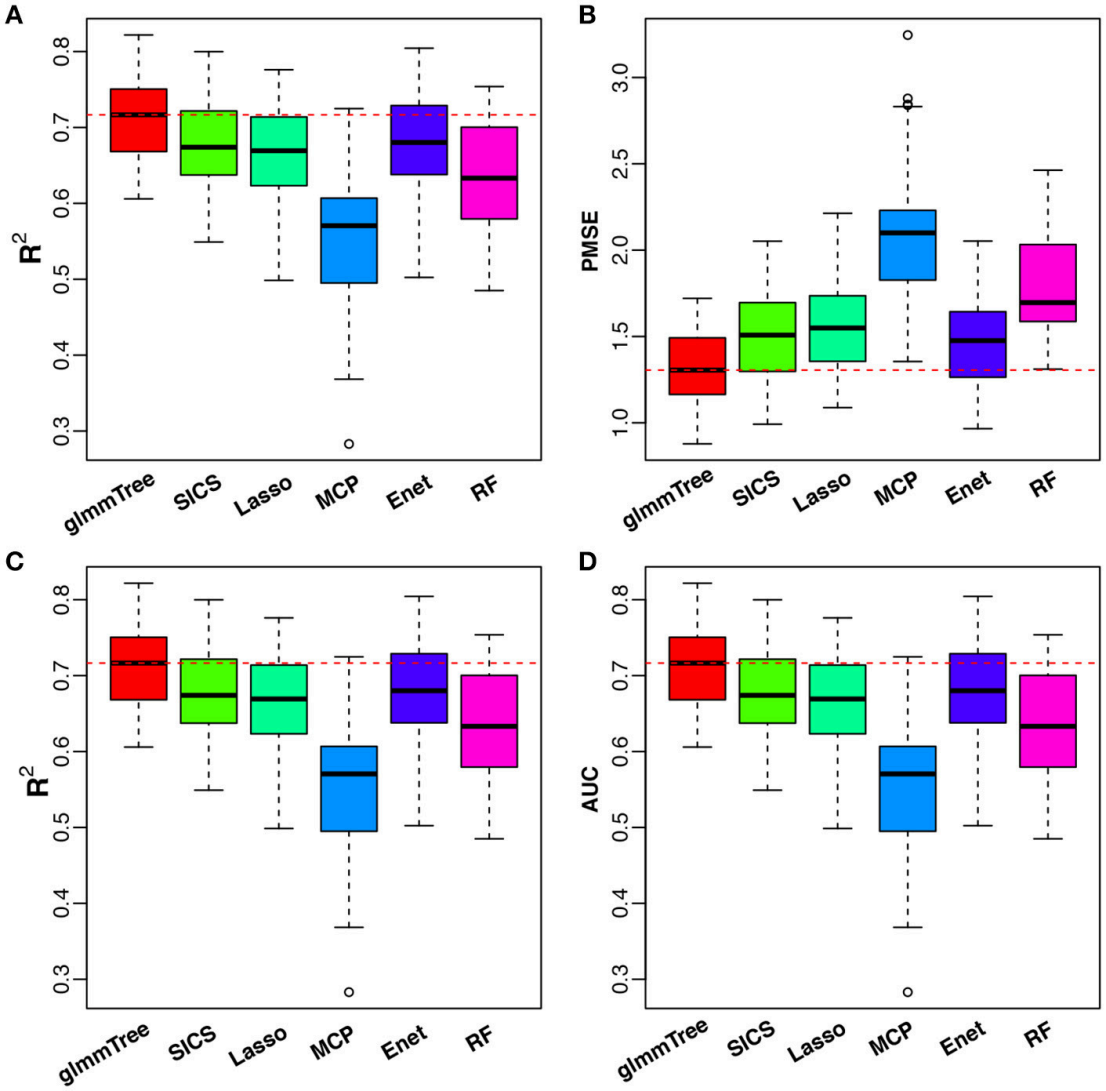
Performance comparison for age prediction. All USA samples are used in continuous age prediction **(A,B)**. Binary prediction is based on the two age groups: baby (0 to 3 years old) and child (3 to 18 years old) **(C,D)**. Red dashed line indicates the median value of various performance measures for glmmTree.

To have an objective evaluation of the prediction performance, we randomly divided the dataset fifty times into five folds: four folds were used for training (with nested CV) and the remaining one fold for testing. *R*^2^ and PMSE were used as metrics for the continuous outcome, while *R*^2^ and AUC were used for the binary outcome. The results are presented in Figure 6. glmmTree achieved the best performance for continuous age prediction as indicated by the highest *R*^2^ and lowest PMSE, followed by SICS and Elastic Net. For baby vs. child prediction, glmmTree still achieved the highest *R*^2^ and AUC, followed by Elastic Net and Random Forest. For child vs. adult prediction, glmmTree and Elastic net achieved the best performance (Figure S6). To verify if the improvement of prediction was significant, we performed paired Wilcoxon signed-rank tests between glmmTree and other methods based on *R*^2^, PMSE and AUC obtained from the fifty random divisions. For continuous age prediction, glmmTree achieved significantly higher *R*^2^, and significantly lower PMSE than other methods (*P*-value < 0.05). For baby vs. child prediction, glmmTree achieved significantly higher AUC than other methods, and significantly higher *R*^2^ than other methods except Elastic Net. For child vs. adult prediction, glmmTree achieved significantly higher AUC and *R*^2^ than other methods except Elastic net. Overall, glmmTree performed the best for both the continuous and binary age outcome on this dataset.

## 5. Discussion

One of the challenges for predictive modeling of microbiome data is the utilization of the phylogenetic tree. As microbiome profiling experiments produce increasingly higher taxonomic resolutions such as strain-level resolution (Truong et al., 2015; Callahan et al., 2016), incorporating the phylogenetic tree information becomes even more important. The phylogenetic tree provides a principled way to pool signals and directs the analysis to the most relevant parameter space, which is essential to counter the “curse of dimensionality.” Previous work indicates that predictive models could benefit from the incorporation of the phylogenetic tree through the use of tree-induced smoothness penalty (Tanaseichuk et al., 2014; Chen et al., 2015; Wang and Zhao, 2017). These models usually induce a sparse solution and are hence efficient to detect sparse and clustered signals. In this work, we propose to utilize the phylogenetic tree to detect dense and clustered signals. This is achieved by assuming the OTU effects as random in a GLMM framework, and that the OTU random effects follow a multivariate normal distribution with the correlation structure defined based on the phylogenetic tree.

We performed comprehensive simulations to investigate the performance of the proposed method at varying cluster sizes, signal densities and taxa abundances. Simulation studies demonstrated that glmmTree favors dense and clustered signals or signals from abundant OTUs, compared to sparse regression models, which has a competitive performance for sparse signals, particularly from those less abundant OTUs. By using a power transformation, glmmTree can capture a wide range of non-linear effects including the biologically relevant scenario where the outcome depends on the presence/absence of the OTUs. Human microbiome studies have frequently found that the species richness (α-diversity) were associated with some phenotypic traits (Le Chatelier et al., 2013). Therefore, capturing the signals on the presence/absence level should not be overlooked.

Our work is closely related to the recently proposed kernel penalized regression framework (Randolph et al., 2015), which provides a theoretic framework to incorporate a variety of extrinsic information, such as phylogeny, into penalized regression models. For microbiome data applications, Randolph et al. (2015) illustrated their method using a kernel-based on UniFrac distances. In our work, we took a further step and optimized the microbiome-based kernel to be capable of capturing clustered signals at various phylogenetic depth as well as accommodating non-linearity. Moreover, our model is based on the generalized linear model, which can handle non-Gaussian outcomes while adjusting for covariates easily.

As the microbiome field matures, more complex study designs such as family and longitudinal studies have been used to study the human microbiome in relation to various clinical and biological variables. These studies are efficient to control potential confounders such as genetics and diet, and are also more powerful than studies based on independent sampling. Although our framework is developed mainly for independent data, it could be modified to accommodate such clustered data by incorporating additional cluster-level random effects. Similar algorithms (i.e., PQL) could be used to fit these multiple random effects model.

The effectiveness of the proposed method depends on the reliability of the phylogenetic tree, which can be very noisy or non-informative. Although our method is robust to tree mis-specification via the tuning parameter ρ, its performance will not be optimal if the tree is severely mis-specified. In this case, other types of kernels without using the tree, such as the radial basis function (RBF) kernel (Shawe-Taylor and Cristianini, 2004), may be more powerful. A composite kernel that combines the tree-based and non-tree-based kernels may increase the robustness of our method for detecting various kinds of dense signals. Furthermore, since the underlying signal structure is unknown for real applications, an ensemble approach incorporating representative prediction methods targeted to different signal structures (e.g., dense vs. sparse) is more likely to provide an even more robust prediction. We leave these extensions as our future work.

## Author contributions

JX analyzed the data, drafted the paper, prepared figures and tables, reviewed drafts of the paper. LC analyzed the data, drafted the paper, prepared figures and tables, wrote the software, reviewed drafts of the paper. SJ revised drafts of the paper. YY contributed to the revision of the paper. XZ contributed substantial expertise to improve the paper and revised the paper. JC conceived and designed the experiments, analyzed the data, wrote the paper, wrote the software, prepared figures and tables.

### Conflict of interest statement

The authors declare that the research was conducted in the absence of any commercial or financial relationships that could be construed as a potential conflict of interest.
