# Supplementary material for: Predictive Modeling of Microbiome Data Using a Phylogeny-Regularized Generalized Linear Mixed Model
Source: Front Microbiol. 2018 Jun 27;9:1391. doi: 10.3389/fmicb.2018.01391 (PMC6030386; doi:10.3389/fmicb.2018.01391)
Supplement: Supplementary file 1 [file Presentation_1.pdf]

Supplementary Material: Predictive modeling of microbiome data using a phylogeny-regularized generalized mixed effects model

1 SUPPLEMENTARY NOTE

1.1 Statistical inference for a continuous outcome

Let $\boldsymbol{\beta} = (\beta_0, \boldsymbol{\beta}_1)^T$ and $\boldsymbol{X}$ be the design matrix including the intercept. The variance-covariance matrix of $\boldsymbol{y}$ is given by

$$\boldsymbol{V}(\sigma_b^2, \sigma_\epsilon^2|\gamma, \rho) = \sigma_b^2 \boldsymbol{f}(\boldsymbol{Z}, \gamma)\boldsymbol{C}(\rho)\boldsymbol{f}(\boldsymbol{Z}, \gamma)^T + \sigma_\epsilon^2 \boldsymbol{I}.$$

Assuming $\boldsymbol{V}$ is known, the Least Squares Estimate (LSE) of estimated coefficients for fixed effects is given by

$$\hat{\boldsymbol{\beta}}(\sigma_b^2, \sigma_\epsilon^2|\gamma, \rho) = (\boldsymbol{X}^T\boldsymbol{V}^{-1}\boldsymbol{X}^{-1})\boldsymbol{X}^T\boldsymbol{V}^{-1}\boldsymbol{y},$$

which is the best linear unbiased estimation (BLUE) of $\boldsymbol{\beta}$. After joint modeling $\boldsymbol{y}$ and $\boldsymbol{b}$, we have

$$E(\boldsymbol{b}|\boldsymbol{y}) = \sigma_b^2 \boldsymbol{C}(\rho)\boldsymbol{f}(\boldsymbol{Z}, \gamma)^T\boldsymbol{V}^{-1}(\boldsymbol{y} - \boldsymbol{X}\boldsymbol{\beta}).$$

Thus, the predicted random effects are

$$\hat{\boldsymbol{b}}(\sigma_b^2, \sigma_\epsilon^2|\gamma, \rho) = \sigma_b^2 \boldsymbol{C}(\rho)\boldsymbol{f}(\boldsymbol{Z}, \gamma)^T\boldsymbol{V}^{-1}(\boldsymbol{y} - \boldsymbol{X}\hat{\boldsymbol{\beta}}),$$

which is the best linear unbiased prediction (BLUP) of $\boldsymbol{b}$.

The estimation of variance components $\sigma_b^2, \sigma_\epsilon^2$ is performed by maximizing the REstricted Maximum-Likelihood method ("REML").

$$\ell_R(\boldsymbol{\beta}(\sigma_b^2, \sigma_\epsilon^2)) = -\frac{1}{2}ln|\boldsymbol{V}| - \frac{1}{2}ln|\boldsymbol{X}^T\boldsymbol{V}^{-1}\boldsymbol{X}| - \frac{1}{2}(\boldsymbol{y} - \boldsymbol{X}\boldsymbol{\beta})^T\boldsymbol{V}^{-1}(\boldsymbol{y} - \boldsymbol{X}\boldsymbol{\beta}) \quad \text{(S1)}$$

Notice that $\boldsymbol{V}$ and $\boldsymbol{\beta}$ are all functions of the variance components $\sigma_b^2, \sigma_\epsilon^2$. It is straightforward to obtain the estimates of the variance components by maximizing the REML. REML is preferred to Maximum Likelihood method ("ML") because it avoids a downward bias of maximum-likelihood estimates of variance components by taking into account the loss in degrees of freedom associated with fixed effects. The optimization problem could be solved by Newton-Raphson algorithm.

1.2 Statistical Inference for a binary outcome

Given a known $\boldsymbol{b}$, y_i is conditionally independent with mean

$$E(y_i|\boldsymbol{b}) = \mu_i^b = \frac{\exp(\beta_0 + \boldsymbol{x}_i^T\boldsymbol{\beta}_1 + f(\boldsymbol{z}_i, \gamma)^T\boldsymbol{b})}{1 + \exp(\beta_0 + \boldsymbol{x}_i^T\boldsymbol{\beta}_1 + f(\boldsymbol{z}_i, \gamma)^T\boldsymbol{b})},$$

and variance

$$var(y_i|\boldsymbol{b}) = \nu(\mu_i^b) = \mu_i^b(1-\mu_i^b).$$

The integrated quasi-likelihood function to estimate $\boldsymbol{\beta}$ and σ_b^2 is defined by

$$\ell_{QL} \approx |\sigma_b^2 \boldsymbol{C}(\rho)|^{-\frac{1}{2}} \int \exp[-\frac{1}{2\phi}\sum_{i=1}^{n} d_i(y_i, \mu_i^{\boldsymbol{b}}) - \frac{1}{2}\boldsymbol{b}^T(\sigma_b^2\boldsymbol{C}(\rho))^{-1}\boldsymbol{b}]d\boldsymbol{b}, \tag{S2}$$

where ϕ is the dispersion parameter ($\phi = 1$ for binary outcome, we thus drop it) , and

$$d_i(y_i, \mu_i^{\boldsymbol{b}}) = -2\int_{y_i}^{\mu_i^{\boldsymbol{b}}} \frac{y_i - u}{\nu(u)}du.$$

The integrated quasi-likelihood function is further approximated by penalized quasi-likelihood (PQL) defined by

$$\ell_{PQL}(\boldsymbol{\beta}, \sigma_b^2|\gamma, \rho) \approx -\frac{1}{2}\sum_{i=1}^{n} d_i(y_i, \mu_i^{\boldsymbol{b}}) - \frac{1}{2}\boldsymbol{b}^T(\sigma_b^2\boldsymbol{C}(\rho))^{-1}\boldsymbol{b}. \tag{S3}$$

To maximize l_{PQL}, a series of liner mixed models could be fitted until convergence. The basic idea is to approximate the generalized mixed model with a working linear mixed model and solve σ_b^2, $\boldsymbol{\beta}$ and $\boldsymbol{b}$ in an iterative way. To be specific, the working outcome $\boldsymbol{y}^* = (y_1^*, \ldots, y_n^*)^T$ is calculated as

$$y_i^* = g(\hat{\mu}_i^{\boldsymbol{b}}) + (y_i - \hat{\mu}_i^{\boldsymbol{b}})g'(\hat{\mu}_i^{\boldsymbol{b}}),$$

where $g(.)$ is the link function (logit function for a binary outcome)), and the working linear mixed model is expressed as

$$\begin{aligned} \boldsymbol{y}^* &= \boldsymbol{X}\boldsymbol{\beta}^* + \boldsymbol{f}(\boldsymbol{Z}, \gamma)\boldsymbol{b} + \boldsymbol{\epsilon}^* \\ \boldsymbol{b}^* &\sim N(0, \sigma_b^2\boldsymbol{C}(\rho)), \boldsymbol{\epsilon}^* \sim N(0, \boldsymbol{W}^{-1}), \end{aligned} \tag{S4}$$

where $\boldsymbol{W}$ is the diagonal matrix of $w_i = \{\nu(\mu_i^{\boldsymbol{b}})(g'(\mu_i^{\boldsymbol{b}}))^2\}^{-1}, i = 1, \ldots, n.$

2 SUPPLEMENTARY FIGURES

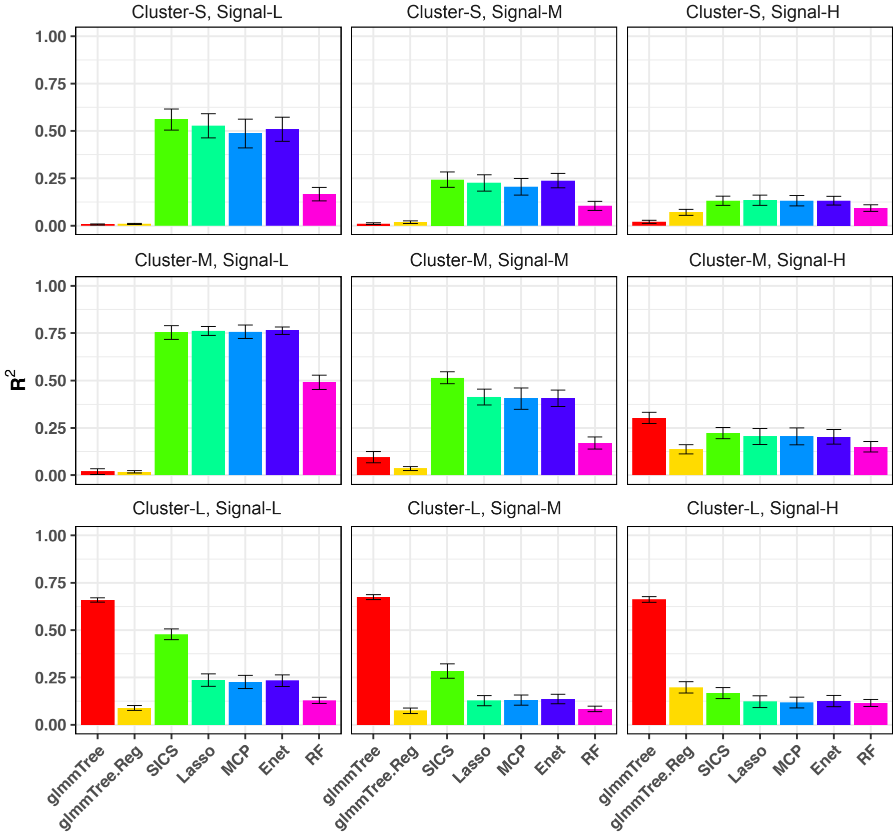

Figure S1: Performance comparison for continuous-outcome simulations when the abundance of associated OTU clusters is low. Cluster-S, -M, and -L represent small, medium and large clusters, and Signal-L, -M and -H represent low, medium and high signal density, respectively.

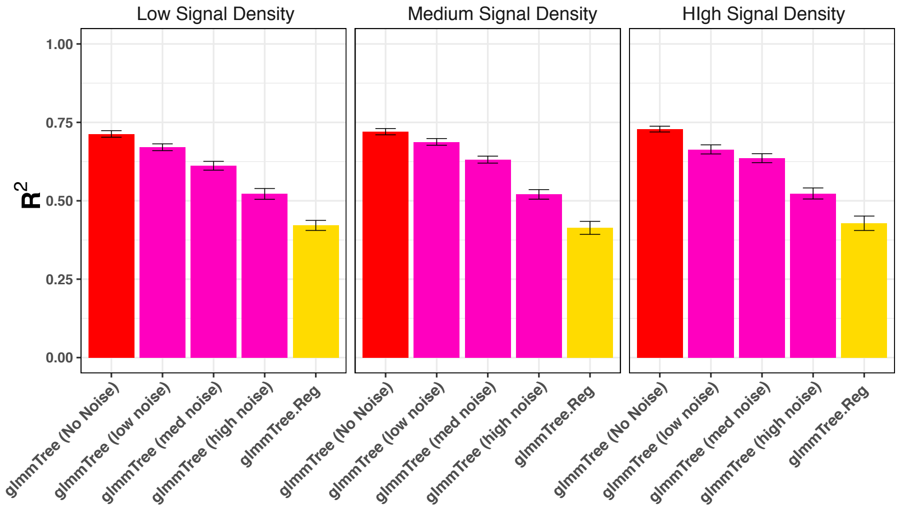

Figure S2: The effect of tree mis-specification. Continuous-outcome data are simulated, and the associated OTU clusters are large and abundant. Different levels of noises are added to the original phylogenetic tree through randomly permuting 25%, 50% and 75% of the columns/rows of the tree-induced distance matrices, representing a "low", "medium" and "high" noise level, respectively.

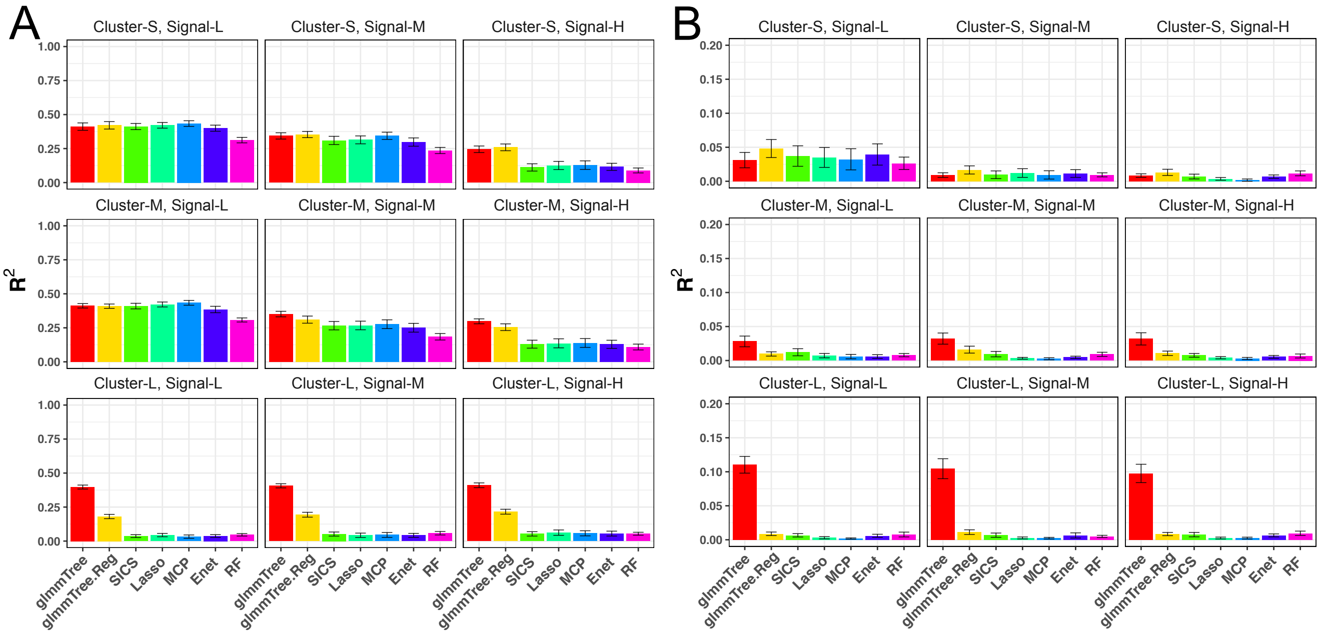

Figure S3: Performance comparison for continuous-outcome simulations under lower signal-noise-ratios. R^2 across different levels of cluster size and signal density are shown. The abundance of associated OTU clusters is chosen to be high, explaining about 50% (A) and 33% (B) of the outcome variability. Cluster-S, -M, and -L represent small, medium and large clusters, and Signal-L, -M and -H represent low, medium and high signal density, respectively.

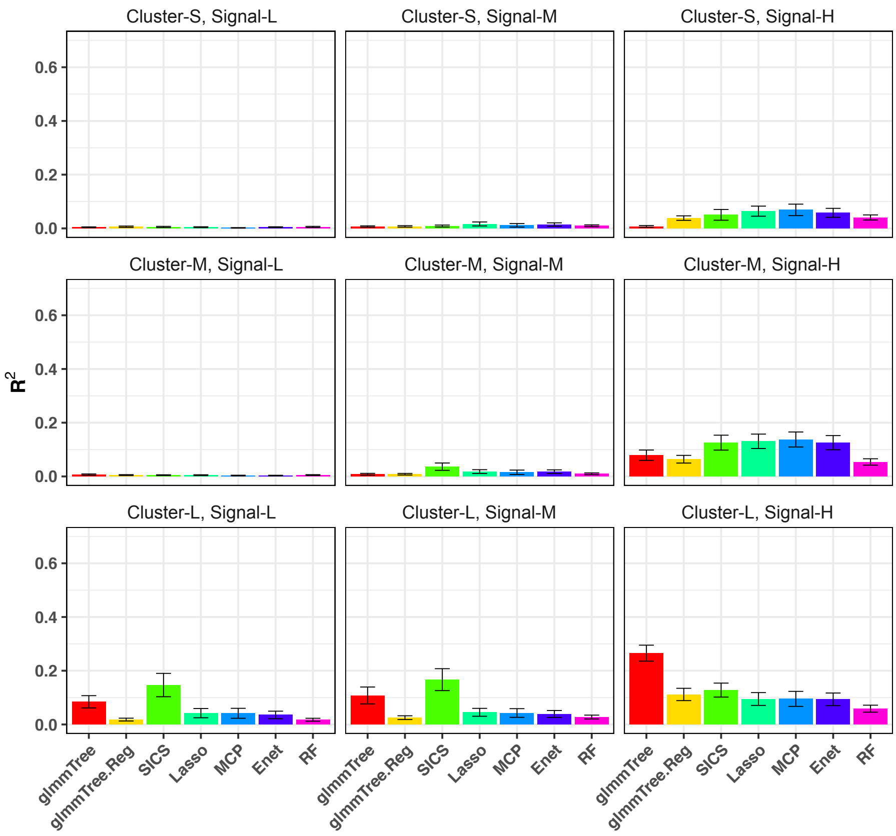

Figure S4: Performance comparison for binary-outcome simulations when the abundance of associated OTU clusters is low. R^2 across different levels of cluster size and signal density are shown. Cluster-S, -M, and -L represent small, medium and large clusters, and Signal-L, -M and -H represent low, medium and high signal density, respectively.

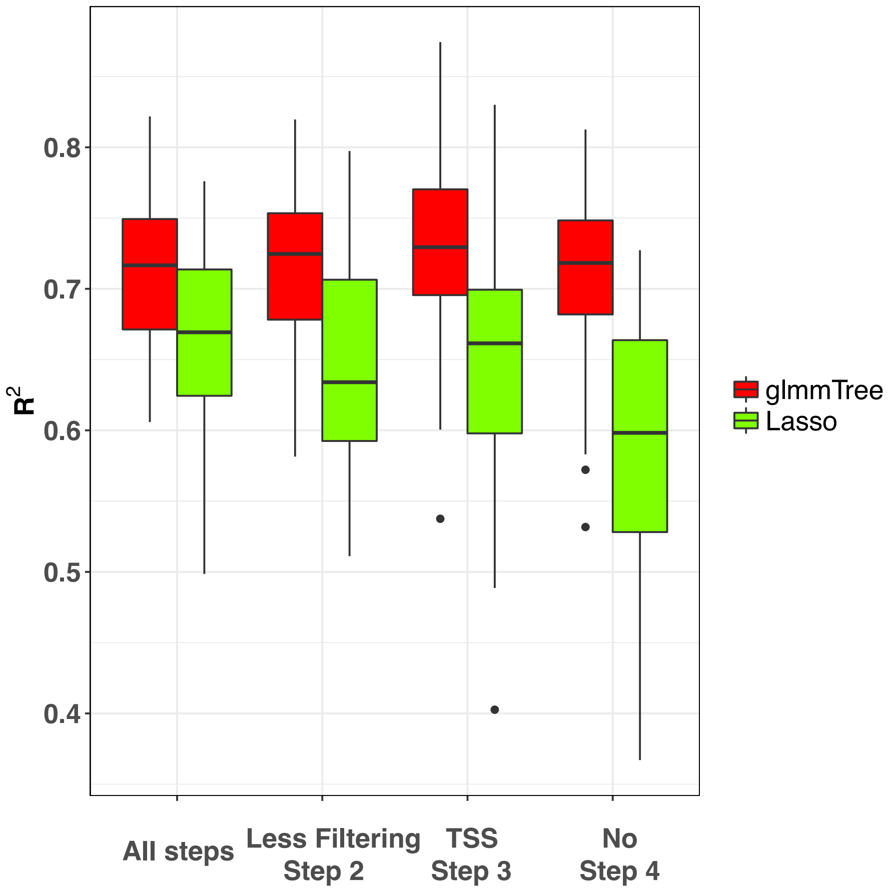

Figure S5: The effect of variation in preprocessing steps on the performance of glmmTree and Lasso for continuous age prediction. We vary the preprocessing steps by imposing a less stringent filter (removing OTUs with median non-zero count less than 5), using TSS normalization in step 3 (total sum scaling, equivalently division by total read counts) or excluding step 4 (no outlier replacement). glmmTree is more robust to these variations while the performance of Lasso decreases.

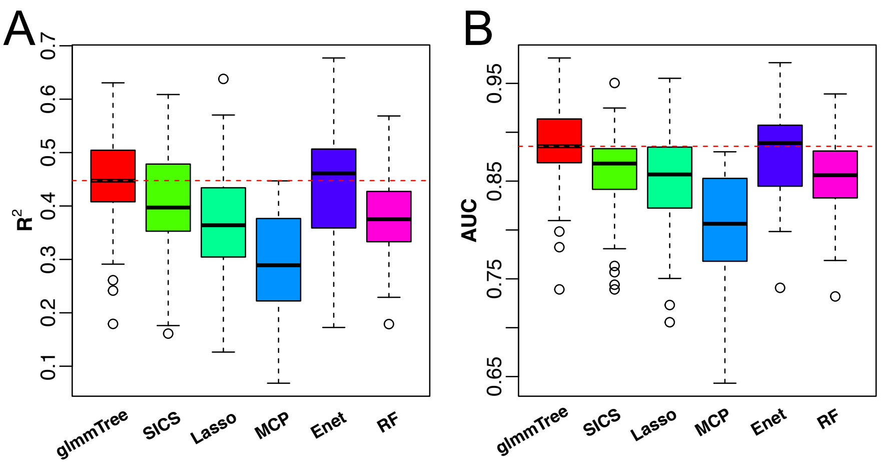

Figure S6: Performance comparison for binary age prediction (child vs. adult). Red dashed line indicates the median of R^2 and AUC for glmmTree.
